# *HIF1A* gene rs10873142 polymorphism is associated with risk of chronic obstructive pulmonary disease in a Chinese Han population: a case–control study

**DOI:** 10.1042/BSR20171309

**Published:** 2018-03-09

**Authors:** Licheng Wang, Yanliang Tang, Ye Chen

**Affiliations:** 1 Department of Intensive Care Unit, Fuyang Hospital of Traditional Chinese Medicine, 4 Guihua Road, Fuyang District, Hangzhou, Zhejiang, China; 2 Department of Pneumology, The Second Affiliated Hospital of Zhejiang Chinese Medical University, Hangzhou, Zhejiang, China

**Keywords:** COPD, HIF1A, single nucleotide polymorphism

## Abstract

Chronic obstructive pulmonary disease (COPD) is a type of obstructive lung disease characterized by long-term poor airflow. Recently, variants in the hypoxia inducible factor 1α (*HIF1A*) gene were found to be associated with COPD risk. The present study aimed to identify whether rs10873142 polymorphism (an intronic polymorphism) in *HIF1A* gene was related to COPD in a Chinese population. We genotyped *HIF1A* gene rs10873142 polymorphism in a case–control study with 235 COPD cases and 548 controls in a Chinese Han population. Odd ratios (ORs) and 95% confidence intervals (CIs) were estimated using the chi-squared (χ^2^) test, genetic model analysis, and stratification analysis. In the genetic model analysis, we found that the TT genotype (TT compared with CC: OR: 1.63; 95% CI: 1.02–2.60; *P*=0.042) and T allele (T compared with C: OR: 1.29; 95%CI, 1.02–1.60; *P*=0.032) showed significant correlation with the risk of COPD. However, in stratification analyses of age, BMI, and forced expiratory volume in 1 s (FEV1)/FEV, we failed to find any association between *HIF1A* gene rs10873142 polymorphism with COPD risk. The present study supports that *HIF1A* gene rs10873142 polymorphism may be associated with increased risk of COPD in a Chinese Han population. To the best of our knowledge, this is the first case–control study uncovering that the *HIF1A* gene rs10873142 polymorphism increases the risk of COPD in a Chinese Han population.

## Introduction

Chronic obstructive pulmonary disease (COPD) is a type of obstructive lung disease characterized by long-term poor airflow. COPD develops as a significant and chronic inflammatory response to inhaled irritants [1]. Although tobacco smoking is the most important risk factor of COPD [2], tobacco smoking differentially affects lung function decline, and not all smokers will develop COPD [3]. The individual response to tobacco smoking and other environmental exposures is affected partly by genetic factors, and the development of COPD is the culmination of the environment acting in concert with a complex array of genetic factors [4].

Chronic hypoxia is a common feature of COPD, which is mainly caused by chronic inflammation. Hypoxia inducible factor-1 (HIF-1) is a transcription factor that acts as principal regulator of oxygen homeostasis, playing a fundamental role in the physiological response to hypoxia. HIF-1 mediates adaptive responses to reduced oxygen availability by regulating gene expression. A critical cell autonomous adaptive response to chronic hypoxia controlled by HIF-1 is reduced mitochondrial mass and/or metabolism. Exposure of HIF-1-deficient fibroblasts to chronic hypoxia results in cell death due to excessive levels of reactive oxygen species [5]. HIF-1 is also involved in intratumoral hypoxia and genetic alterations that inactivate tumor suppressor genes or activate oncogenes. HIF-1 is a heterodimer composed of two subunits: hypoxia inducible factor -1α (HIF-1α) (HIF1A) and HIF-1β. HIF-1β is a constitutive core protein, whereas expression of HIF-1α is regulated by oxygen concentration. HIF-1α subunit is subject to ubiquitination and proteasomal degradation, a process that is inhibited under hypoxic conditions [5]. Recent data indicate that HIF-1α plays a major role in some cancers [6–9]. Furthermore, multiple lines of evidence demonstrated that hypoxia was involved in the development of chronic inflammatory processes [10] and factor hypoxia-1 inducible acted as a regulator of the development of inflammation [11,12]. Nevertheless, the possible involvement of genetic alterations in *HIF1A* gene has not been well studied in the COPD inflammation process. Therefore, we hypothesized that HIF1A takes a part in the development and prognosis of COPD.

The *HIF1A* gene is located in the chromosome 14q23.2 and has 16 exon counts. According to dbSNP database, rs10873142 polymorphism is located in the intron region of *HIF1A* gene. Although rs10873142 is an intronic single nucleotide polymorphism (SNP), we suppose that this SNP may be in linkage disequilibrium with other SNPs to regulate *HIF1A* gene transcription and protein translation. We also searched 1000 Genomes Browser (https://www.ncbi.nlm.nih.gov/variation/tools/1000genomes/) and found the minor allele frequency (MAF) of C allele in rs10873142 polymorphism is 0.386. The MAF of C allele in rs10873142 polymorphism in the cases was 0.32 in a Spanish study [13] and 0.418 in a Chinese from Hainan province [14]. Two studies [13,14] have investigated the association between *HIF1A* gene rs10873142 polymorphism and COPD risk, and found no significant association. To date, there is no gene association study of this abovementioned SNP amongst the Chinese Han population in Eastern China. Thus, the aim of the present case–control study was to investigate whether *HIF1A* gene rs10873142 polymorphism is associated with the risk and development of COPD in a Chinese Han population from Zhejiang province (Eastern China).

## Methods

A total of 783 participants (235 COPD cases and 548 controls) were consecutively recruited from the Second Affiliated Hospital of Zhejiang Chinese Medical University, between May 2013 and September 2016. COPD was newly diagnosed according to the criteria established by the National Heart, Lung and Blood Institute/World Health Organization Global Initiative for Chronic Obstructive Lung Disease (GOLD) [15]. None of the patients had a previous history of other cancers, chemotherapy, or radiotherapy. Participants were chosen without restrictions of age, sex, or disease stage. The healthy controls were free of COPD and recruited from the same institutions during the same time period. They were frequency matched to the COPD cases based on sex and age (±9.69 years). A detailed questionnaire related to smoking habits was completed for each patient and control by a trained interviewer. Informed consent was obtained from all the patients and controls prior to their participation. The protocol for the present study was approved by the Ethics Committee of the Second Affiliated Hospital of Zhejiang Chinese Medical University (Hangzhou, Zhejiang, China).

### DNA extraction and genotyping

For the selection of SNP required to genotype in *HIF1A* gene, we reviewed previous case–control studies of COPD [13,14]. We then selected one SNP, rs10873142. To investigate the *HIF1A* gene rs10873142 polymorphism, all study participants provided 2 ml of peripheral blood in EDTA tubes and stored at −80°C until use. The peripheral blood samples were collected from the individuals in the morning. DNA was extracted by using the QIAamp DNA Blood Mini Kit (Qiagen, Hilden, Germany). SNP genotyping was performed using a custom-by-design 48-Plex SNP scan™ kit (Genesky Biotechnologies Inc., Shanghai, China), which was based on double ligation and multiplex fluorescence PCR. This kit was developed according to patented SNP genotyping technology by Genesky Biotechnologies Inc., which was based on double ligation and multiplex fluorescence PCR.

### Statistical analysis

The SPSS 11.0 statistical software (SPSS Inc., Chicago, IL, U.S.A.) was used for statistical analysis. In all the analyses, the lower frequency allele was coded as the ‘risk’ allele. All *P*-values presented in the present study were two sided, and we used *P*<0.05 as the cut-off value for statistical significance. An exact test was used to assess the variation in each SNP frequency from Hardy–Weinberg equilibrium (HWE) in the control subjects. The demographic and clinical characteristics of study participants were evaluated by using the chi-squared test. Odds ratios (ORs) and 95% confidence intervals (CIs) were used to estimate the association between *HIF1A* gene polymorphisms and risk of COPD by logistic regression analyses.

## Results

### The characteristics of the study population

The characteristics of COPD cases and healthy controls in the present study were summarized in Table 1. There were no significant differences in age, sex, and smoking status between two groups.

**Table 1:** Characteristics of the study population

Variables	Patients (*n*=235)	Controls (*n*=548)	*P*
Age (years)	66.73 ± 9.62	66.32 ± 9.69	0.586
Sex (male/female)	188/47	430/118	0.630
Body mass index	26.00 ± 3.35	25.31 ± 3.53	0.011
FEV1/FVC (%)	58.42 ± 6.50	86.68 ± 6.37	<0.001
Smoking status (no/yes)	94/141	236/312	0.426

Abbreviations: FEV1, forced expiratory volume in 1 s; FVC, forced vital capacity.

### Association between *HIF1A* gene rs10873142 polymorphism and COPD risk

The frequencies of the genotypes for rs10873142 polymorphism in cases and controls were shown in Table 2. Genotype distributions for rs10873142 polymorphism in the controls conformed to the HWE. In addition, we found the risk of COPD in individuals with TT genotype was 1.6-times higher than that of individuals with CC genotype (TT compared with CC: OR: 1.63; 95% CI: 1.02–2.60; *P*=0.042). The T allele of rs10873142 polymorphism was associated with the increased risk of COPD (T compared with C: OR: 1.29; 95% CI: 1.02–1.60; *P*=0.032). Furthermore, the significant association was observed in the allelic model (*P*=0.032), but not in the recessive model (*P*=0.108) and dominant model (*P*=0.063). No relationships between genotypes of H1F1A rs10873142 polymorphism and clinical characteristics (age, BMI, and forced expiratory volume in 1 s (FEV1)/FEV) were obtained (Table 3). Reviewed data extracted from recent review [16] was presented in Table 4, which could attract more citations and to better plan future studies.

**Table 2:** Logistic regression analysis of associations between HIF1A rs10873142 polymorphism and risk of COPD

Genotype	Cases* (*n*=235)		Controls* (*n*=548)		OR (95% CI)	*P*
	*n*	%	*n*	%		
CT compared with CC	111/80	47.2/34.0	246/227	44.9/41.4	1.28 (0.91–1.80)	0.153
TT compared with CC	39/80	16.6/34.0	68/227	12.4/41.4	**1.63 (1.02–2.60)**	0.042
TT + CT compared with CC	150/80	63.8/34.0	314/227	57.3/41.4	1.36 (0.98–1.87)	0.063
TT compared with CT + CC	39/191	16.6/81.2	68/473	12.4/86.3	1.42 (0.93–2.18)	0.108
T compared with C	189/271	40.2/57.7	382/700	34.9/63.9	**1.29 (1.02–1.60)**	0.032

Bold values are statistically significant (*P*<0.05).

* The genotyping was successful in 230 cases and 541 controls.

**Table 3:** The clinical and biochemical characteristics of HIF1A rs10873142 polymorphism amongst two groups

	Patients (*n*=230)				Controls (*n*=541)			
	CC (*n*=80)	CT (*n*=111)	TT (*n*=39)	*P*	CC (*n*=227)	CT (*n*=246)	TT (*n*=68)	*P*
Age (years)	66.75 ± 9.56	67.28 ± 9.77	65.51 ± 9.68	0.618	66.54 ± 9.73	66.32 ± 9.69	65.57 ± 9.67	0.771
BMI (kg/m^2^)	26.53 ± 3.47	25.78 ± 3.19	25.54 ± 3.37	0.197	25.50 ± 3.65	25.12 ± 3.49	25.46 ± 3.29	0.477
FEV1/FEV	58.79 ± 6.29	58.11 ± 7.07	58.83 ± 5.46	0.722	87.06 ± 6.12	86.11 ± 6.50	87.76 ± 5.14	0.090

Bold values are statistically significant (*P*<0.05).

**Table 4:** Reviewed data

Polymorphism name	Population	Sample size	Association with diseases
rs10873142	Caucasians	1375	(+) Coronary artery disease [22]
	Caucasians and Melanoderm	6118	(+) Idiopathic osteonecrosis of the femoral head in men [23]
	Asians	376	(−) Acute myocardial infarction and frequent intradialytic hypotension [24]
	Caucasians	346	(−) Early-onset pre-eclampsia [17]
	Caucasians	297	(−) Lung cancer [18]
	Asians	601	(−) COPD [14]
rs41508050	Caucasians	297	(−) Lung cancer [18]
rs2301113	Asians	601	(−) COPD [14]
rs11549465	Caucasians	36	(+) Lung cancer [25]
	Asians	48	(+) COPD [19]
	Caucasians and African Americans	233	(+) Maximal oxygen consumption [26]
	Caucasians	297	(−) Lung cancer [18]
	Asians	285	(−) Lung cancer [27]
	Asians	154	(−) Lung cancer [28]
rs11549467	Asians	48	(+) COPD [19]
	Caucasians	297	(−) Lung cancer [18]
	Asians	285	(−) Lung cancer [27]
	Asians	154	(−) Lung cancer [28]
rs199775054	Asians	47	(−) Lung cancer [29]
rs113182457 rs60361955	Caucasians	36	(+) Lung carcinoma [25]
rs10645014	Caucasians	297	(−) Lung cancer [18]

(+) Observed association, (−) no association.

## Discussion

In the present study, we found that *HIF1A* gene rs10873142 polymorphism was associated with the increased risk of COPD in a Chinese Han population. TT genotype and T allele of rs10873142 polymorphism showed a significant correlation with the risk of COPD. Stratified analyses did not obtain any associations between this SNP and age, BMI, and FEV1/FEV.

*HIF1A* gene rs10873142 polymorphism T allele was previously demonstrated to be associated with a higher transcriptional activity and increased angiogenesis. Inherited susceptibility to increased HIF1α expression resulting in the up-regulation of angiogenic genes may mediate a protective effect in normal pregnancy and pregnancy complicated by late onset pre-eclampsia in a Sinhalese population [17]. Konac et al. [18] found that *HIF1A* gene rs10873142 polymorphism is not related with lung cancer susceptibility in a Turkish population. Putra et al. [19] found that HIF1A gene polymorphisms (C1772T and G1790A) are not associated with COPD development. In a subsequent study form China, Wei et al. [20] obtained a significant association between G1790A polymorphism and COPD risk, but not C1772T. Similar results were also replicated in the study of Yu et al. [21] from Shandong province, China. However, two above-referred loci (C1772T and G1790A) are not the SNP we investigated. Maybe rs10873142 polymorphism is in linkage disequilibrium with these loci, which needs further studies to validate whether these SNPs regulate HIF1A gene transcription and protein translation. Moreover, a recent review conducted by Gladek et al. [16] showed that HIF1A gene rs10873142 polymorphism is associated with the risk of coronary artery disease with stable exertional angina [22] and idiopathic osteonecrosis of the femoral head in men [23], while not associated with acute myocardial infarction and frequent intradialytic hypotension [17], early-onset pre-eclampsia [17], lung cancer [18], and COPD [14]. More data in this review included are shown in Table 4.

Two studies have explored the association between *HIF1A* gene rs10873142 polymorphism and COPD risk previously [13,14]. Ding et al. [14] first conducted a case–control study in China to identify susceptibility alleles of *HIF1A* gene rs10873142 polymorphism for COPD, analyzing 200 cases and 401 controls. They provided evidence that *HIF1A* gene rs10873142 polymorphism was not associated with the risk of COPD [14]. Baz-Davila et al. [13] also performed an overall analysis between this SNP and COPD risk in a Spanish cohort with a total of 189 COPD cases and 536 controls. No association was obtained in their studies [13]. In this study, we found TT genotype or T allele carriers was associated with an increased risk of COPD. We cannot rule out the possibility that the results of this study may be attributed to false positivity due to the small sample size. This study consisted of 235 COPD cases and 548 controls. Above studies [13,14] and the present study reported conflicting results obviously. These conflicting findings may be attributed to some factors, including clinical heterogeneity, different sample sizes, and ethnic differences. It is noteworthy that COPD cases of previous studies [13,14] did not match the controls about age, which may affect the final findings of these studies. In addition, the study by Ding et al. [14] included two nationalities (Han and Li) in Hainan province, while the present study only contained one nationality (Han) in Zhejiang. We hypothesized that it may explain the different results of the present study and the study by Ding et al. [14]. Moreover, we did not find any relationship between *HIF1A* gene rs10873142 polymorphism (age, BMI, and FEV1/FEV) and clinical or lung function parameters in the present study.

The potential limitations of the present study merits careful consideration. First, this was a hospital-based case–control study; therefore, a selection bias was unavoidable and the subjects are not fully representative of the general population. Second, the polymorphisms investigated, which were based on functional considerations, may not offer a comprehensive view of the genetic variability of HIF1A. Third, the present study only investigated one SNP of *HIF1A* gene, but other SNPs of *HIF1A* gene are warranted. Fourth, the present study did not investigate whether this SNP regulates *HIF1A* gene transcription and protein translation. Fifth, the association between *HIF1A* gene rs10873142 polymorphism and clinical symptoms need to be evaluated in future studies. Sixth, the sample size is relatively small, thus the present study may be underpowered. Finally, the rs10873142 polymorphism is located in the intron 8 of *HIF1A* gene. The functional significance of HIF1A rs10873142 polymorphism is unclear. The association between HIF1A rs10873142 polymorphism and COPD risk may reflect linkage disequilibrium with another potentially functional variant or closely linked susceptibility gene, which need be further confirmed. Exon regions of *HIF1A* gene should be tested in future COPD association studies due to the significant association of exon region with *HIF1A* gene transcription and protein translation.

In summary, the present study verifies that *HIF1A* gene rs10873142 polymorphism may be associated with the risk of COPD in a Chinese Han population. Further studies with larger sample sizes are warranted to verify whether *HIF1A* gene rs10873142 polymorphism is associated with COPD risk.

## Abbreviations

BMI: body mass index
CI: confidence interval
COPD: chronic obstructive pulmonary disease
FEV1: forced expiratory volume in 1 s
HIF1: hypoxia inducible factor-1
HIF1A: hypoxia inducible factor-1α
HWE: Hardy–Weinberg equilibrium
MAF: minor allele frequency
OR: odds ratio
SNP: single nucleotide polymorphism
